# Doxorubicin and Quercetin Double Loading in Modified MCM-41 Lowered Cardiotoxicity in H9c2 Cardioblast Cells In Vitro

**DOI:** 10.3390/bioengineering10060637

**Published:** 2023-05-24

**Authors:** Christina Voycheva, Teodora Popova, Marta Slavkova, Virginia Tzankova, Denitsa Stefanova, Diana Tzankova, Ivanka Spassova, Daniela Kovacheva, Borislav Tzankov

**Affiliations:** 1 Department of Pharmaceutical Technology, Faculty of Pharmacy, Medical University of Sofia, 1000 Sofia, Bulgaria; hvoycheva@pharmfac.mu-sofia.bg (C.V.); tpopova@pharmfac.mu-sofia.bg (T.P.); mslavkova@pharmfac.mu-sofia.bg (M.S.); 2 Department of Pharmacology, Pharmacotherapy and Toxicology, Faculty of Pharmacy, Medical University of Sofia, 1000 Sofia, Bulgaria; vtzankova@pharmac.mu-sofia.bg (V.T.); denitsa.stefanova@pharmfac.mu-sofia.bg (D.S.); 3 Department of Pharmaceutical Chemistry, Faculty of Pharmacy, Medical University of Sofia, 1000 Sofia, Bulgaria; d.tsankova@pharmfac.mu-sofia.bg; 4 Institute of General and Inorganic Chemistry, Bulgarian Academy of Sciences, 1113 Sofia, Bulgaria; ispasova@svr.igic.bas.bg (I.S.); didka@svr.igic.bas.bg (D.K.)

**Keywords:** MCM-41 nanoparticles, carboxylic functionalization, doxorubicin, quercetin, cardiotoxicity, double loading

## Abstract

Background: One of the therapeutic limitations of the use of doxorubicin (DOX) as an anticancer drug is its cardiotoxicity. Its hydrophilicity also causes difficulties in achieving sustained release. The simultaneous delivery with the well-known natural antioxidant quercetin could ameliorate its cardiotoxicity. Thus, the main aim of this work is to study the potential of carboxylated and non-carboxylated mesoporous silica MCM-41 nanoparticles for double loading of the hydrophilic doxorubicin hydrochloride and hydrophobic quercetin (Q) in one nanocarrier with a modified release pattern to reduce the cardiotoxic side effects of doxorubicin in vitro. Methods: The methods included the modification of MCM-41, single and double loading of modified and non-modified MCM-41, physicochemical characterization, in vitro release tests and kinetic study, and in vitro cell viability studies. Results: Doxorubicin and quercetin were successfully double-loaded with encapsulation efficiency (EE) of 43 ± 4.1% and 37 ± 4.5%, respectively, in native MCM-41. The post-synthetic carboxylation led to 49 ± 4.3% EE (DOX) and 36 ± 4.0% (Q) and double lowering of the cardiotoxicity on H9c2 (IC_50_ = 5.96 µm). Sustained release profiles over 72 h were achieved. Conclusions: A successful procedure was proposed for the efficient double loading of a hydrophilic drug and a hydrophobic drug. The carboxy-modified double-loaded nanosystems demonstrate a decreased in vitro cardiotoxicity of doxorubicin and can be considered as a potential chemotherapeutic formulation.

## 1. Introduction

Doxorubicin (DOX) is an anthracycline obtained from Streptomyces peucetius and is readily soluble in water [1]. It is clinically applicable to treat hematological and solid tumors [1,2,3]. The main mechanisms of antiproliferative activity of DOX in tumor cells include free radical generation, inhibition of topoisomerase II, and DNA intercalation [4]. However, the wide use of DOX is limited due to its low specificity and extensive cardiotoxicity, which results in increased mortality risk [5]. Many studies on the mechanisms of toxicity have shown that the overproduction of reactive oxygen species (ROS) plays an important role in its toxicity to the myocardium [6,7]. The main cause of DOX cardiotoxicity is mitochondrial oxidative stress and topoisomerase 2β-mediated DNA damage [8,9]. DOX tends to accumulate in the cardiolipids of the inner mitochondrial membranes and eventually leads to myocardial cell death [10]. Overcoming doxorubicin-induced cardiotoxicity requires the identification of specific targets in the endogenous antioxidant defense system. One potential possibility is the nuclear erythroid 2-related factor (Nrf2). Its suppression enhances DOX-induced ROS formation, cardiomyocyte necrosis and cardiac dysfunction [11]. Therefore, Nrf2 can potentially be considered a specific target for treating DOX-induced cardiac dysfunction. Natural compounds, such as baicalein [12,13], genistein [14], neferin [15] and quercetin, improve the myocardial antioxidant status and might ameliorate DOX-induced cardiac damage in different in vitro and in vivo models. 

Quercetin is a plant flavonoid with low aqueous solubility (0.00215 g/L at 25 °C for anhydrous quercetin and 0.00263 g/L at 25 °C for quercetin dihydrate) [16]. It possesses various pharmacological properties, including antioxidant, anticancer, anti-inflammatory, antihypertensive, antiobesity, vasodilator, antihypercholesterolemic and antiatherosclerotic activities [17,18]. Of particular interest are its cardioprotective and antioxidant activities. Their main mechanism includes direct “trapping” of ROS and subsequent prevention of cellular damage [19,20]. Other protection mechanisms are through modulation of gene expression or interaction with different enzyme systems. Suppression of lipid peroxidation processes also plays a role in the process [21,22]. In addition, quercetin has an anti-inflammatory effect [23,24] and shows its own antitumor activity [25]. Therefore, the co-administration of DOX and polyphenols with antioxidant activity such as quercetin can be assumed to limit the chemotherapeutic cardiotoxicity while maintaining its cytostatic properties. In a recent in vivo experimental study, the cardioprotective effects of different polyphenols (included in micelles) were investigated. It was evident that a reduction in DOX-induced cardiotoxicity together with the chemosensitizing effect of their simultaneous delivery with DOX can be achieved [26]. In this study, however, only the natural polyphenols were loaded into micelles while DOX was non-encapsulated but applied concurrently. We assumed that the double loading of DOX and quercetin on a suitable drug delivery nanosystem for controlled release would have several advantages from a technological and biopharmaceutical point of view in regard to a reduction in the cardiotoxicity.

Unfortunately, active substances with different physicochemical properties such as quercetin and doxorubicin are typically difficult to simultaneously incorporate in nanosized drug delivery systems (DDSs) [27]. These difficulties arise from the nanosystems’ specificity and often from the needed complex chemical reaction to achieve the desired conjugation [28,29]. However, mesoporous silica nanoparticles can provide a solution to overcome this issue, by a modification in the loading procedure [29]. This would provide the application of already available techniques and exploit all the advantages of mesoporous silica nanoparticles. 

Mesoporous silica nanoparticles (MSNs) are widely used inorganic nanocarriers because of their higher aqueous dispersion stability, excellent biocompatibility, and in vivo biodegradability [30]. MCM-41 nanoparticles are a type of MSN with mesopores structured in a hexagonal array [31] and advantageous characteristics, such as tunable pore size from 2 to 5 nm and high pore volume and surface area. Based on these characteristics, they offer a high drug-loading capacity compared to other nanocarriers [32]. Moreover, their very small pore size is a prerequisite for a high level of amorphization and an increase in drug solubility [33,34]. The high number of free silanol groups on their outer and inner pore surfaces makes their functionalization a convenient strategy for modifying drug delivery [35]. Attaching a carboxyl moiety is possible either during or after the mesoporous particles’ synthesis. Usually, the post-synthesis –COOH modification is a two-step process, including a previous amino functionalization [36,37]. This specific modification of the silanol groups could be beneficial regarding the loading efficiency [37] and modification of the drug release pattern [38,39]. The chemical functionalization offers new binding sites, and drugs can be loaded by means of covalent and non-covalent interactions [40,41]. Nevertheless, the post-production carboxylation of MSNs could lead to the pitfall of possible pore blockage, thus limiting the effective drug loading [37,42]. In the case of double loading, the encapsulation efficiency can be decreased due to competition for free volumes or functional groups [27]. The simultaneous loading of drugs with different properties (hydrophilic and hydrophobic) can be achieved by a relatively simple method of sequential adsorption in different solvents [29]. Therefore, careful and tailored modification of MSNs is required to achieve co-delivery in chemotherapy.

Nowadays, DOX is mainly administered intravenously due to its lower oral bioavailability (around 5%). Unfortunately, the parenteral administration has many disadvantages associated with the painful application as well as large fluctuations in drug plasma concentration. The recent tendency in anticancer therapy is to find a technological approach to replace intravenous administration with peroral [43]. 

The present study aimed to develop a peroral drug delivery system based on post-synthetic carboxy-modified mesoporous silica nanoparticles (MCM-41-COOH) double-loaded with the hydrophilic doxorubicin and the hydrophobic quercetin while maintaining acceptable encapsulation efficiency. The simultaneous release of the drugs from the proposed MSN-based drug delivery system was intended to result in a reduction in the in vitro DOX-induced cardiotoxicity on the H9c2 cardioblast cell line. In vitro cytotoxicity evaluation of empty MCM-41 and MCM-41-COOH particles was also performed to ensure their safety. 

## 2. Materials and Methods

### 2.1. Materials 

The active pharmaceutical ingredients (APIs), namely doxorubicin hydrochloride and quercetin, as well as empty MCM-41 nanoparticles, 3-aminopropyltriethoxysilane (APTES), succinic anhydride, ethanol, anhydrous toluene, McCoy’s 5A medium, heat-inactivated horse serum (HSDH), L-glutamine and colchicine, were purchased from Sigma Aldrich (Darmstadt, Germany). An Alamar Blue assay kit was obtained from PerkinElmer, Waltham, MA, USA. All the materials used were of high chemical grade and used with no need for additional purification. Deionized water was prepared by the ion exchange method.

### 2.2. Methods

#### 2.2.1. Carboxylation of MCM-41

**Figure 1:**
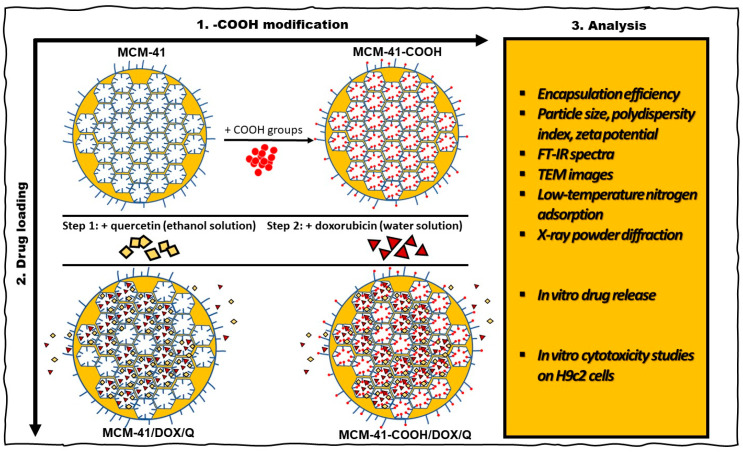
Schematic preparation and characterization of MCM-41/DOX/Q and MCM-41-COOH/DOX/Q.

Functionalization of MCM-41 with carboxylic groups (Figure 1) included a two-stage procedure—intermediate amination followed by carboxylation. First, the native MCM-41 nanoparticles were dried (2 h at 120 °C) to remove the adsorbed water. Then, the amination was carried out by mixing MCM-41 nanoparticles with 3-aminopropyltriethoxysilane (APTES) in ethanol for 5 h at 50 °C under continuous stirring. The obtained nanoparticles were centrifuged and subsequently washed twice with ethanol and water and afterward dried.

Further carboxylation of the already prepared amino-modified MCM-41 nanoparticles was performed, assuming they possessed 2 wt% amino content. The carboxylation followed a previously described procedure [44]. In brief, the second stage included the addition of succinic anhydride (6.6 mmol) to the dispersion of the amino-modified MCM-41 in anhydrous toluene at 60 °C for 24 h. Finally, the carboxylated MCM-41 nanoparticles (MCM-41-COOH) were dried for 6 h by vacuum evaporation at 25 °C temperature.

#### 2.2.2. Quercetin and Doxorubicin Loading

Quercetin (Q) loading on both MCM-41 and carboxylated MCM-41 (MCM-41-COOH) was performed by incubation of 100 mg dried nanoparticles in 5 mL (1 mg/mL) ethanol solution of Q (4 h, 37 °C) with subsequent evaporation of the solvent and drying. Samples were washed with water, collected by centrifugation (15 min, 15,000 rpm) and named MCM-41/Q and MCM-41-COOH/Q.

Doxorubicin was loaded using 1 mg/mL aqueous solution of DOX following the same procedure as in the case of quercetin loading at room temperature. The obtained samples were named MCM-41/DOX and MCM-41-COOH/DOX.

The loading of the active substances was achieved by the solvent-impregnation method [45]. 

Dual drug loading (Figure 1) was performed using a two-step process as proposed by Liu et al. [29] including initial loading with an ethanol solution of quercetin. After the first loading procedure, the solvent was completely removed, followed by a secondary loading of the systems with doxorubicin in an aqueous solution.

Double-loaded nanoparticles were obtained using the already loaded MCM-41/Q and MCM-41-COOH/Q. A sample of 100 mg of them was incubated in 5 mL of a 1 mg/mL aqueous solution of DOX. The water was further evaporated, and the resulting nanoparticles—MCM-41/DOX/Q and MCM-41-COOH/DOX/Q—were washed with ethanol, collected by centrifugation (15 min, 15,000 rpm) and dried.

#### 2.2.3. Encapsulation Efficiency

Determination of the encapsulation efficiency (*EE*%) for DOX and Q from modified and non-modified MCM-41 nanoparticles was carried out by calculation of the difference between the total amount of the corresponding APIs used for loading (*API_TOTAL_*) and the amount found in the supernatants (*API_NATANT_*) according to the following equation:

$$EE\%=\frac{API_{TOTAL}-API_{NATANT}}{API_{TOTAL}}\;100$$

The supernatant consisted of the combined washings of the corresponding dispersions after centrifugation. The assays of both APIs were performed by HPLC (modular HPLC system UltiMate Dionex 3000 SD, equipped with diode array detector, Thermo Fisher Scientific, Waltham, MA, USA) using a Kromasil C18 (250 × 4.6 mm, 5 μm) column. A methanol–acetonitrile–phosphate buffer with pH 3.8 in the ratio 19:29:52 (*v*/*v*/*v*), a flow rate of 1.0 mL/min and an injection volume of 15 μL was chosen as a mobile phase. The detection wavelengths for quercetin and doxorubicin were set to 256 nm and 480 nm, respectively, by using Chromeleon 7.2 SR3. The amounts of Q and DOX were determined based on predetermined calibration curves in the range of 0.2–100 µg/mL (R2 ≥ 0.9999) using standard solutions.

#### 2.2.4. Particle Size, Polydispersity Index, Zeta Potential

Particle size, polydispersity index and zeta potential of empty and loaded nanoparticles were determined using a Zetasizer Nano ZS (Malvern Instruments, Malvern, UK). The samples were dispersed in deionized water at 25 °C and measured at a scattering angle of 90°.

#### 2.2.5. FTIR

Any possible interaction between the nanocarriers and active substances was investigated. Attenuated total reflection infrared (ATR-FTIR) spectra over the spectral region from 400 to 4000 cm^−1^ were recorded with Nicolette 400 spectrometer (Thermo Fisher Scientific, USA).

#### 2.2.6. TEM

Transmission electron microscopy (TEM) images were recorded using a JEOL JEM 2100 HR STEM (200 KV; point resolution 0.23 nm).

#### 2.2.7. Low-Temperature Nitrogen Adsorption

Texture parameters were evaluated by low-temperature (77.4 K) nitrogen adsorption in a NOVA 1200e instrument (Quantachrome Instruments, Boynton Beach, FL, USA). Adsorption–desorption isotherms used for the determination of the specific surface areas (S_BET_), total pore volumes (V_t_) and average pore diameters (D_av_) were estimated at p/p_0_ ≈ 1. 

#### 2.2.8. X-ray Powder Diffraction

Wide-angle X-ray powder diffraction patterns were collected in the range 5–80° 2θ with a step of 0.02° 2θ on a Bruker D8 Advance diffractometer (Cu Kα radiation, LynxEye detector). Small-angle diffraction patterns were collected in the 0.5–5° 2θ range, step 0.02° 2θ, using an adjustable knife edge.

#### 2.2.9. In Vitro Drug Release

In vitro drug release studies were performed using an incubator shaker (Julabo Shake Temp SW23, Merck KGaA, Darmstadt, Germany) at 37 °C under a 100 rpm shaking rate. Each sample (10 mg) (including the free APIs as control samples) was placed into a 10 mL 0.1 M hydrochloric acid solution with pH 1.2, and phosphate buffer solution (PBS) with pH 5.0 or pH 6.8 was the acceptor phase. Samples were withdrawn at appropriate time intervals and centrifuged at 15,000 rpm for 15 min. HPLC was used to determine the concentrations of released APIs following the procedure described in Section 2.2.3. The time for 50% of released APIs (T_50_) was used to characterize the drug release. To ensure accurate and reproducible results, all the experiments were performed in triplicates, and the difference was evaluated with ANOVA at a significance level of *p* < 0.05.

#### 2.2.10. Release Kinetics

Equations of known theoretical models describing release kinetics, including zero-order, first-order, Higuchi and Korsemeyer–Peppas, were used. The data from the release studies were fitted to the models in order to determine the doxorubicin and quercetin release mechanism.

#### 2.2.11. Cell Culturing and In Vitro Cell Viability Studies

The rat cardioblast cell line (H9c2) was acquired from the European Collection of Cell Cultures (ECACC, Salisbury, UK). The cells were cultured in a medium prepared with DMEM low-glucose basic media supplemented with 10% heat-inactivated fetal bovine serum(FBS) and 2 mM L-glutamine. The cardioblast cells were seeded in 96-well plates at a cell density of 1 × 10^4^ cells/well and incubated overnight at 37 °C, 5% CO_2_ and high humidity (Esco CelCulture CO₂ Incubator, CCL-170B-8-IVF, Esco Micro Pte. Ltd., Singapore). The culture’s medium was replaced at a time interval of 1–2 days. After 24 h of incubation, the cells were treated with MCM-41; MCM-41-COOH aqueous solution of free DOX (0.01–20 μM); Q (0.021–41.4 μM in DMSO); and the developed single- or double-loaded particles MCM-41/DOX, MCM-41/Q, MCM-41/DOX/Q, MCM-41-COOH/DOX, MCM-41-COOH/Q and MCM-41-COOH/DOX/Q with corresponding concentrations of DOX and Q. Cell viability was assessed by MTT test [46] after 48 h of incubation with the test solutions. 

#### 2.2.12. Statistical Analysis

GraphPad Prism 6 GraphPad Software, Inc., La Jolla, CA, USA) was used for the statistical analysis. The results were expressed as mean values ± SD (*n* = 8). One-way ANOVA followed by Dunnett’s post hoc test was applied. In vitro experiments were carried out in triplicate.

## 3. Results and Discussion

### 3.1. Encapsulation Efficiency

The results presented in Table 1 show the encapsulation efficiency of the different single- and double-loaded MSNs. The encapsulation efficiency for DOX in the bare nanoparticles is lower compared to that in the functionalized nanocarriers (*p* < 0.05). The loading is mainly due to hydrogen bonding and electrostatic interactions as shown by the FTIR data. Comparable encapsulation efficiencies are reported in the literature [47,48,49]. Many studies have been performed in order to improve the DOX loading through surface modification of MCM-41 [37,50,51]. The introduction of a carboxylic group is a successful strategy in this direction. A study performed by Zaharudin et al. [37] showed the EE% of 45% for the hydrophilic drug gemcitabine to be the highest in –COOH-modified MSNs. The mechanism of adsorption for this drug is similar to the one for DOX as it contains an amine group. The amino groups of DOX take part in electrostatic interactions with the carboxylic groups of the nanoparticles [51,52]. Another study by Sanots et al. [52] demonstrated slightly higher loading percentages of DOX in the MCM-41 (52.4%) and MCM-COOH (57.6%) they synthesized. The difference with the results in our experiment most likely arise from the different size and surface area of the used MSNs. Furthermore, Santos et al. apply a gating agent simultaneously with the API loading, and thus no DOX is lost during the washing of the nanocarriers. Nevertheless, the same tendency is observed, namely increased encapsulation efficiency in the carboxylated MSNs in comparison to the non-modified nanoparticles. In another study, it was proven that DOX tends to self-aggregate on the silica surface [53]. This would hinder the entrance deep inside the pores. 

The quercetin loading in either of the nanocarriers is lower than the loading of doxorubicin. Our results are in accordance with the results presented by Zaharudin et al. [37]. The slight differences which are observed could be due to the different mesoporous silica nanoparticles they used. The data of the current work are also confirmed by the study of Ugazio et al. [54]. In our study, pure ethanol was used as it was shown that the highest loadings are achieved in this solvent [55]. Quercetin is predominantly loaded by hydrogen bonds with silanol groups (see FTIR data) which [37] are abundant in the non-functionalized nanoparticles as compared to the carboxy-modified ones. Similar data have been shown by Berlier et al. [56] and Tzankov et al. [57].

The different dissolutions of the APIs led us to employ a sequential loading approach. Quercetin was loaded in the initial stage, as it is hydrophobic, and its optimal loading takes place in anhydrous conditions [55]. After the ethanol was removed and the particles were dispersed in an aqueous doxorubicin solution, the already loaded quercetin remained immobilized in the mesopores due to its practical insolubility in water.

**Figure 2:**
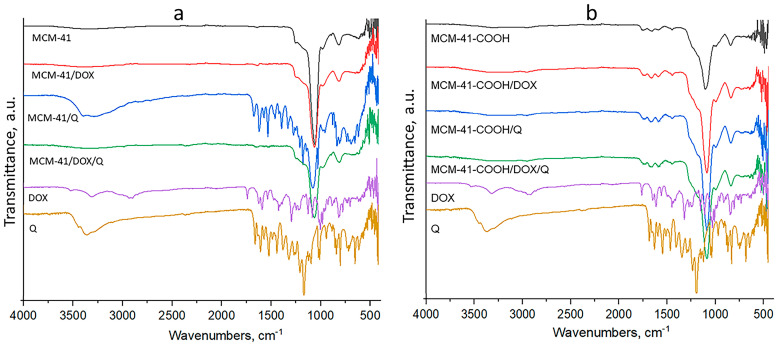
FTIR spectra of (**a**) non-modified MCM-41 nanoparticles, free DOX and Q for comparison, and (**b**) carboxy-modified MSNs, free DOX and Q for comparison.

The double loading did not result in a decrease in the encapsulation efficiency for either of the drugs. Furthermore, we did not aim for the maximum degree of loading (i.e., filling the entire available free volume) but the maximum use of the free surface of the particles for possible interactions of the drug with the carrier. As can be seen (Table 2), the free volume of non-functionalized particles changed from 1.24 cm^3^ to 0.88 cm^3^ after loading with quercetin and decreased to 0.47 cm^3^ after the addition of doxorubicin. The trend is similar for the carboxylated particles—after loading with quercetin, the volume decreased from 0.61 cm^3^ to 0.34 cm^3^, and after loading with doxorubicin, it reached 0.12 cm^3^. It can be seen that in both types of systems, even after double loading, there is residual load capacity; i.e., the pores are not blocked. In regard to the FTIR results (Figure 2), we hypothesize that the loading of quercetin occurs mainly through the formation of hydrogen bonds between the drug substance and the surface-located free silanol groups on the carrier. The results presented by us show that the loading of doxorubicin in the non-functionalized particles is related mainly to physical incorporation in the free volumes of the mesopores. At the same time, in the carboxylated particles, it is more related to electrostatic interactions between the amino group of the drug substance and the carboxyl groups of the carrier. This fact also explains the higher EE of doxorubicin in the carboxylated carrier (Table 1). The EE of quercetin remains almost the same in the dual-loaded particles. This is mainly due to the proposed modified loading method and the immobilization of the Q in the DOX aqueous solution. Particle size (Table 1) and XRD data show that there are no drug crystals on the particles’ surface. Therefore, we consider that the active substances found on the surface of the particles are in a small amount and are loaded according to the already described mechanisms.

The possibility of double loading two APIs and maintaining high encapsulation efficiency is probably due to different loading mechanisms. Their different solubilities and sequential loading allow maximal exploitation of the nanocarrier. The data of FTIR analysis confirm this assumption. The low-temperature nitrogen adsorption also showed a more pronounced decrease in the specific surface area and pore volume in the case of double loading (see Table 2). 

### 3.2. Particle Size, Polydispersity Index, Zeta Potential

The zeta-potential measurements of all models demonstrate negative values (Table 1). As shown, the zeta potential is more negative for the carboxylated particles than for the non-modified ones. This observation can indicate the efficient carboxylic functionalization of the silica carriers, in agreement with IR analysis, as upon deprotonation, the –COOH groups provide further negative charges. Although the zeta potential decreased, it remained above the critical value (±20 mV) for all the samples, maintaining nanoparticle stability. Furthermore, the results of particle size determination of single- and double-loaded samples indicate that even though carboxylated samples have a higher particle size, the polydispersity index decreased upon modification of MCM-41, which is evidence of the lack of agglomeration. The size of the prepared systems ranges between 415 nm and 616 nm (Table 1). The carboxy-modified nanoparticles show larger values due to the surface functionalization. The results of other studies showed that the size could be suitable for their peroral administration [58,59]. 

### 3.3. FTIR

MCM-41 nanoparticles were characterized by an intense band of materials at 1042 cm^−1^, which was due to the asymmetric stretching vibration of silica structure (Si–O–Si). In addition, the band at 962 cm^−1^ corresponded to the surface Si–O groups [60] (Figure 2a). 

The stretching vibrations of O-H and N-H bonds in the area of 3300–3550 cm^−1^ are provoked by DOX. The peaks at 1615 cm^−1^ and 1580 cm^−1^ correlate with the phenol group, 1524 cm^−1^ with the aromatic ring, 1410 cm^−1^ with N-H stretching, 1230 cm^−1^ with C-N stretching and 993 cm^−1^ with alcohol groups and were confirmed with literature data [42]. Furthermore, a significant lowering of the intensities of the characteristic bands of DOX was observed in the spectrum of MCM-41/DOX, which is evidence of the successful encapsulation of doxorubicin. On the other hand, the loading of MCM-41 with DOX did not result in the shifting of characteristic bands of the silanol groups, probably due to the physical entrapment of DOX in the pores of the nanocarriers. 

Quercetin shows a characteristic band at 3360 cm^−1^ provoked by OH, and OH bending of the phenol group was detected at 1410 cm^−1^ [43]. 

In the spectra of quercetin-loaded MCM-41 nanoparticles, the bands mentioned above are retained but with a lower intensity, indicating the loading of Q into the pores. On the other hand, the increased H-bond formation between silanol MCM-41 groups and -OH groups of quercetin results in the shifting of the OH characteristic band to 3406 cm^−1^. The successful carboxylation of MCM-41 is proved by the significant shifting of silanol group bands to 1070 cm^−1^ and 946 cm^−1^ and the appearance of the stretching vibration of carboxylic groups at 1550, 1620 cm^−1^ (ionized) and 1730 cm^−1^ (nonionized) [44]. The loading of DOX in MCM-41-COOH shifts the characteristic bands of the carboxylate ions by 10 cm^−1^, which gives us reason to assume that the active substance is bound by electrostatic interaction between its amino groups and the carboxyl groups of the modified particles. Loading of Q in carboxylated MCM-41 leads to an increased number of H bonds, proved by the shifting of characteristic bands of the nonionized carboxyl groups to 1716 cm^−1^. In the spectra of the double-loaded MCM particles, the presence of doxorubicin was proven with its characteristic bands at 1620 cm^−1^ and 1584 cm^−1^ (phenol ring), and the characteristic pick of quercetin at 1410 cm^−1^ was detected, but both were lower in intensity. On the other hand, the characteristic bands of nonionized carboxyl groups shifted to 1714 cm^−1^ because of the participation in hydrogen bonds with the hydroxyl groups of quercetin, which is indirect evidence for its successful loading.

### 3.4. TEM

**Figure 3:**
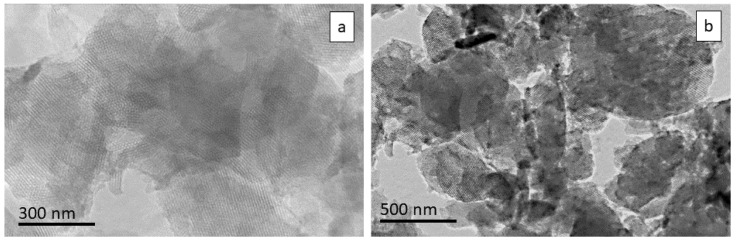
TEM images of COOH-modified MCM-41: unloaded (**a**) and double-loaded (**b**).

TEM was used as a technique to assess size and porous structure. The pictures of both –COOH-modified samples (empty—Figure 3a; double-loaded—Figure 3b) show a size that is in good accordance with DLS analysis. The well-defined porous structure is maintained after carboxy modification and drug loading.

### 3.5. Low-Temperature Nitrogen Adsorption

**Figure 4:**
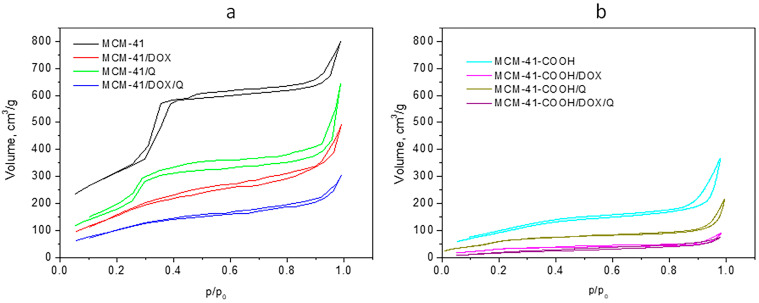
Nitrogen adsorption–desorption isotherms of (**a**) MCM-41 and (**b**) MCM-41-COOH series.

Low-temperature nitrogen adsorption measurements were performed to characterize the porous structure of the empty MCM-41, carboxylated MCM-41 carriers, and single- and double-loaded nanoparticles. Figure 4 shows both series’ adsorption–desorption isotherms, and Table 2 presents the texture parameters obtained. As can be seen from Figure 4, the adsorption isotherm of the carrier MCM-41 is of IV type, according to the IUPAC classification, representing a well-defined and homogeneous porous structure of a mesoporous material with a complex H4–H1 type hysteresis loop evidencing the bi-disperse structure of the material. The single and the double loading of the nanoparticles MCM-41/DOX, MCM-41/Q and MCM-41/DOX/Q (Figure 4a) do not substantially change the porous structure of the carrier and preserve the IV type of the isotherm. However, as expected, the drug loading leads to a decrease in both specific surface areas (S) and total pore volumes (Vt) that reflects the average pore diameter also due to the partial filling or blocking of the pores. The values for MCM-41/DOX are smaller than those for MCM-41/Q due to the loading of the larger molecule doxorubicin. The decrease is more pronounced with the double-loaded nanoparticles MCM-41/DOX/Q as a reflection of the effect of loading of two drugs. 

The carboxylation of the MCM-41 leads to a sharp decrease in the specific surface area and total pore volume and an increase in the average pore diameter due to the filling of part of the pores of MCM-41 (Figure 4b). The texture of the single- and double-loaded nanoparticles follows the expected decreased values due to additional filling or blocking of the pores by the drugs. The isotherms are also of type IV, but the shape of the isotherms and the hysteresis loops are not as defined in the MCM-41 series. This is an indication of less pore ordering of the nanoparticles on the carboxylated MCM-41-COOH.

### 3.6. X-ray Powder Diffraction

**Figure 5:**
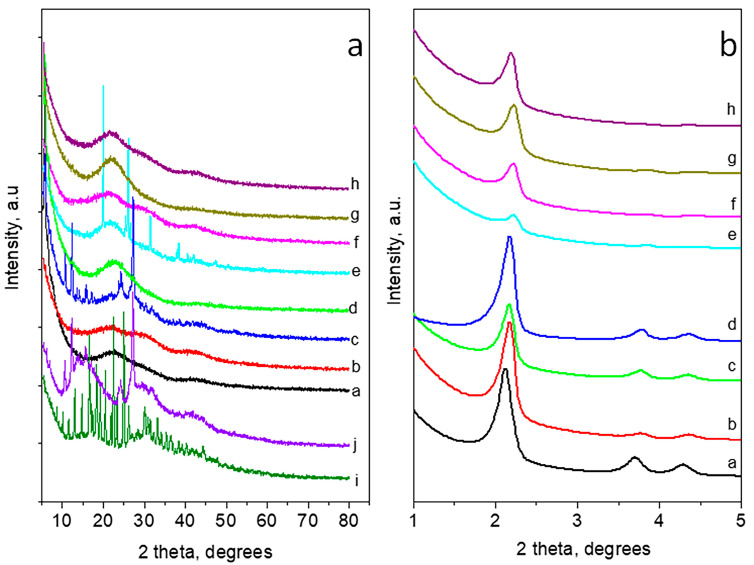
Wide-angle XRD patterns (**a**) and small-angle XRD patterns (**b**) of a—MCM-41, b—MCM-41/DOX, c—MCM-41/Q, d—MCM-41/DOX/Q, e—MCM-41-COOH, f—MCM-41-COOH/DOX, g—MCM-41-COOH/Q, h—MCM-41-COOH/DOX/Q, i—doxorubicin, j—quercetin hydrate.

Wide- and small-angle powder diffraction patterns of the carriers and loaded MCM-41 and carboxylated MCM-41 (MCM-41-COOH) nanoparticles are presented in Figure 5a,b, respectively. The XRD pattern of doxorubicin consists of a large number of sharp peaks at scattered angles below 50° 2 theta, showing high crystallinity [61]. The pattern of quercetin corresponds to its hydrated form [57]. The wide-angle XRD part of MCM-41 presents a typical pattern of amorphous silica. As for the MCM-41 loaded series, the DOX-loaded and double DOX/Q-loaded nanoparticles show amorphous patterns also. The XRD pattern of Q-loaded nanoparticles comprises a superposition of an amorphous part due to the MCM-41 and peaks corresponding to quercetin dihydrate. Small-angle parts (up to 5° 2 theta) of MCM-41 and MCM-41 loaded series exhibit three peaks corresponding to (100), (110) and (200) of hexagonal pore ordering (SG p6m). The loading of the drugs on MCM-41 does not substantially affect the long-range pore ordering. The wide-angle part of the XRD of carboxylated MCM-41, along with the amorphous silica part, shows some peaks of crystalline succinic acid due to the preparation procedure. The patterns of single- and double-loaded nanoparticles of the carboxylated series are amorphous. The decrease in the intensities of the peaks in the small-angle part of XRD patterns for all carboxylated samples is an indication of the deterioration of the long-range pore ordering. These results confirm the data from the low-temperature nitrogen adsorption.

### 3.7. In Vitro Drug Release

Since our systems are intended to be used for peroral administration, we performed dissolution studies in standard media with pH 1.2 and 6.8. A medium with pH 5 was chosen to simulate the environmental conditions in tumor cells (see Table 3) [62].

As a cationic drug (pKa = 8.3), DOX release from all the samples was faster into media with pH 1.2 and pH 5.0 (lower T_50_ values) compared to pH 6.8 [62,63]. The release behavior of quercetin from obtained samples in different pH media was totally different. The presence of the phenolic hydroxyl group is reflected in pH-dependent solubility, leading to an increase in Q release with increasing pH [64]. That is the reason why the obtained systems release Q faster (lower T_50_) into pH 6.8 and 5.0, compared to pH 1.2.

**Figure 6:**
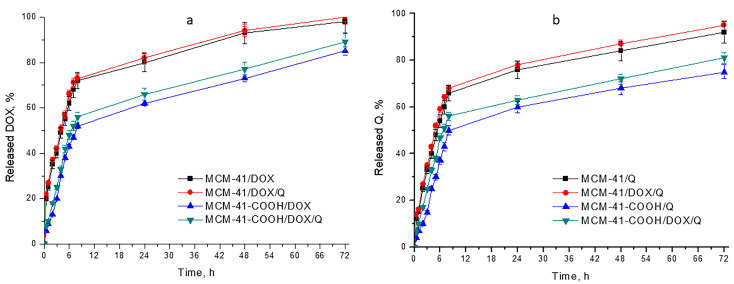
In vitro release profiles of (**a**) DOX and (**b**) Q from the non-modified and carboxy-functionalized MCM-41 in a release medium with pH = 5.0; mean ± SD (n = 3).

This opposite behavior of the two APIs gave us the reason to choose the compromise dissolution medium of pH 5.0, in which both have satisfactory solubility. This also mimics the solid tumor microenvironment [65]. Therefore, the release profiles are shown in pH 5, and it is used in the kinetic studies. As shown in Figure 6, the loading of the APIs onto mesoporous silica shows sustained drug release in comparison to the free APIs (data for free APIs not shown). This could be a prerequisite for lowering the systemic toxicity of the chemotherapeutic, as lower concentration levels will be maintained over time. Comparing the release behavior of quercetin and doxorubicin (Table 3), it can be concluded that in all the models, the release of doxorubicin (T_50_ between 3.60 and 7.45 h) was faster than that of quercetin (T_50_ between 4.68 and 8.22 h) due to the lower solubility of the latter. However, these differences in the release rate between DOX (Figure 6a) and Q (Figure 6b) are not so pronounced for carboxylated samples, where the amount of DOX released is nearly the same as the amount of Q released. This atypical behavior of carboxylated nanoparticles is due to the formation of electrostatic interactions between the amino groups of DOX and the carboxyl groups of the modified particles, which lead to the drug release being sustained (proved by IR spectra analysis). 

The overall effect of the double loading of both non-modified and modified samples on drug release is expressed as a slight increase in the API amount released (the difference is not significant, *p* > 0.05) compared to single-loaded samples. The dissolution process from such systems occurs after medium penetration into the meso-channels and the dissolution of an active substance. The dissolution of one of the APIs leads to the release of new free spaces for diffusion, resulting in a faster release rate. 

The faster release of APIs from the double-loaded non-modified nanoparticles (MCM-41/DOX/Q) is more significant for doxorubicin, which is in accordance with the fact that the quercetin was firstly loaded into MCM-41 nanoparticles, taking place in the spaces of the inner pores. In contrast, DOX was loaded later and probably surrounded the outer pores of the nanoparticles. On the other hand, in the case of the modified double-loaded sample (MCM-41-COOH/DOX/Q), the amounts of DOX and Q released are nearly the same, and comparison cannot be accurately made since, on one hand, DOX interacts with the carboxyl groups of MCM-41-COOH, leading to slower release rate, but on the other hand, it should be released faster since it is predominantly situated in the outer pores. 

Overall, it can be concluded that the double loading of DOX and Q does not lead to significant changes in the release rate of these APIs, compared to single-loaded samples, and therefore these systems have great potential for anticancer therapy. 

### 3.8. Kinetic Models 

In order to describe the release kinetics of our systems in a medium with pH 5.0, we applied four types of mathematical models—zero-order, first-order (√t), Higuci and Korsmeyer–Peppas (K–P).

As can be seen from the results presented in Table 4, the release behavior of all the modified and non-modified MCM-41 samples, regardless of the API loaded, fitted the best with K-P release kinetics, as proven by the highest R^2^ value obtained for this mathematical model. This model describes the anomalous diffusion of drug release. The value of n (release exponent) is used to characterize the release mechanism [66,67]. In the case of a sphere matrix, n = 0.43 indicates Fickian diffusion, 0.43 < n < 0.85 corresponds to anomalous non-Fickian diffusion, and n ≥ 0.85 corresponds to CASE II relaxation-controlled erosion diffusions [68]. 

The higher value of the release exponent (n is between 0.43 and 0.85) for most of the systems indicated highly anomalous diffusion behavior. However, carboxylated samples with DOX (MCM-41-COOH/DOX and MCM-41-COOH/DOX/Q) had an n value equal to 0.43 (Fickian value). Therefore, such systems release DOX according to normal Fickian diffusion [69]. This phenomenon could be explained by the formation of electrostatic interactions between the amino groups of DOX and the carboxyl groups of the modified nanoparticles (proved by IR), which affected the mechanism of DOX release.

### 3.9. In Vitro Cytotoxicity Studies on H9c2 Cells

The in vitro safety profile of empty mesoporous silica nanocarriers MCM-41 and modified MCM-41-COOH was evaluated in rat cardioblast H9c2 cells. Both MCM-41 and MCM-41-COOH were not cytotoxic to H9c2 cells, since no decrease in cell viability was detected in the concentrations from 0.134 to 270 µg/mL. The results from the toxicity evaluation, performed by MTT test, indicated that both types of empty mesoporous silica nanoparticles did not directly alter mitochondrial function, confirming a lack of in vitro cytotoxicity and a promising in vitro safety profile of the empty nanosystems on rat cardioblast cells. 

As mentioned previously, DOX administration is associated with severe cardiotoxicity, which could limit its broad therapeutic applications. Several mechanisms are involved in cardiotoxicity, including increased oxidative stress and lipid peroxidation [70]. Quercetin is a naturally derived active substance with proven antioxidative properties that might provide beneficial effects in reducing the cardiotoxicity of DOX. Thus, our next goal was to evaluate the in vitro cytotoxicity effects of MCM-41 and carboxy-modified MCM-41-COOH mesoporous silica nanoparticles double-loaded with DOX and quercetin on H9c2 cardioblast cells. The effects were compared to those of a free non-loaded combination of DOX and Q, as well as to the effects of quercetin single-loaded in both mesoporous silica nanoformulations (Table 5). 

As expected, significant cardiotoxicity of free DOX on H9c2 cells was observed after 48 h of incubation (IC_50_ = 0.972 μM). Interestingly, we found that single loading of DOX in non-carboxylated MCM-41 (MCM-41/DOX) decreased the cytotoxicity on H9c2 cells, compared to free DOX (IC_50_ =1.636 μM). The single loading of DOX in modified carboxylated silica nanoparticles MCM-41-COOH/DOX decreased its cardiotoxicity even more (IC_50_ = 2.756 μM), compared to non-carboxylated single-loaded MCM-41/DOX.

Our expectations that the double loading of Q together with DOX might have beneficial effects in decreasing the cytotoxic effects of DOX on cardioblast H2c2 cells were proved by the following findings: double loading of DOX and quercetin in both non-carboxylated and carboxylated mesoporous silica nanoparticles (MCM-41/DOX/Q and MCM-41-COOH/DOX/Q) statistically significantly increased the cell viability of cardioblast H9c2 cells (IC_50_ = 3.028 μM and IC_50_ = 5.964 μM, respectively). In vitro estimated cardioprotective effects of double-loaded MCM-41/DOX/Q and especially of carboxylated MCM-41-COOH/DOX/Q were much higher than those of the free combination of non-encapsulated active compounds (DOX + Q) and those of single-loaded Q (when used in the corresponding concentrations). Thus, we found that the development of carboxyl-modified double-loaded MCM-41 with doxorubicin and quercetin shows promising results in decreasing the in vitro cardiotoxicity of DOX. This approach might be beneficial for resolving the safety issues with the drug, which is widely used in oncology practice.

## 4. Conclusions

The present study showed the possibility of post-synthetic MCM-41 carboxy-modification to load a hydrophilic drug and a hydrophobic drug together, with increased encapsulation efficiency for doxorubicin. Both non-modified and modified samples exhibit sustained drug delivery which would provide persistent exposure of the cancer cells to the APIs. Furthermore, the release profiles are synchronized, which is a prerequisite for an improved effect. The simultaneous delivery of quercetin together with doxorubicin showed lower cardiotoxicity in the in vitro cell viability study. This effect is even more pronounced for the post-synthetic carboxylated mesoporous nanoparticles. Thus, the proposed system can be considered as a potential chemotherapeutic tool for dual drug delivery. Once the safety of the application is confirmed, further studies could provide more information on the anticancer effect and the stability of the loaded APIs.

## Figures and Tables

**Table 1:** Results of the DLS analysis of empty, single-loaded and double-loaded modified and non-modified MCM-41 nanoparticles and encapsulation efficiency (EE) for the loaded samples (mean ± SD; n = 3).

	DOX-EE (% ± SD)	Q-EE (% ± SD)	Z-Potential, mV	PDI	Average Size, nm
MCM-41	-	-	−24.0	0.36	415 ± 18
MCM-41/DOX	41 ± 4.9%	-	−22.7	0.45	480 ± 14
MCM-41/Q	-	38 ± 3.9%	−21.9	0.50	486 ± 6
MCM-41/DOX/Q	43± 4.1%	37 ± 4.5%	−22.1	0.56	490 ± 12
MCM-41-COOH	-	-	−29.1	0.31	580 ± 16
MCM-41-COOH/DOX	48 ± 3.7%	-	−26.6	0.35	603 ± 24
MCM-41-COOH/Q	-	36 ± 4.4%	−24.9	0.36	606 ± 15
MCM-41-COOH/DOX/Q	49 ± 4.3%	36 ± 4.0%	−25.1	0.42	616 ± 7

**Table 2:** Texture parameters of MCM-41 and MCM-41-COOH series.

Sample	S_BET_ m^2^/g	V_t_ cm^3^/g	D_av_ nm
MCM-41	1139	1.24	4.0
MCM-41/DOX	658	0.76	4.6
MCM-41/Q	860	0.88	4.0
MCM-41/DOX/Q	420	0.47	4.5
MCM-41-COOH	380	0.61	6.4
MCM-41-COOH/DOX	222	0.17	3.1
MCM-41-COOH/Q	267	0.34	5.1
MCM-41-COOH/DOX/Q	75	0.12	6.6

**Table 3:** Time for 50% DOX or Q release (T_50_) from modified and non-modified samples in different media (mean ± SD, n = 3).

	pH 1.2	pH 5.0	pH 6.8
	T_50_ of DOX, h		
MCM-41/DOX	3.33 ± 0.17	3.92 ± 0.32	5.1 ± 0.31
MCM-41/DOX/Q	3.06 ± 0.25	3.6 ± 0.24	4.68 ± 0.24
MCM-41-COOH/DOX	6.33 ± 0.37	7.45 ± 0.29	9.69 ± 0.16
MCM-41-COOH/DOX/Q	5.16 ± 0.36	6.07 ± 0.16	7.89 ± 0.31
	**T_50_ of Q, h**		
MCM-41/Q	6.76 ± 0.24	5.2 ± 0.33	4.41 ± 0.22
MCM-41/DOX/Q	6.08 ± 0.19	4.68 ± 0.21	3.98 ± 0.18
MCM-41-COOH/Q	10.67 ± 0.28	8.22 ± 0.09	6.98 ± 0.31
MCM-41-COOH/DOX/Q	9.05 ± 0.43	6.97 ± 0.14	5.91 ± 0.22

**Table 4:** Kinetic model fitting results for DOX and Q release in phosphate buffer release medium with pH 5.0.

	Zero-Order Q_t_ = Q_0_ − k_0_t		First-Order lnQ_t_ = lnQ_0_ − k_1_t		Higuchi Qt = k_H_t^1/2^		Korsmeyer–Peppas (K–P) $\frac{Mt}{M\infty }=k.t^{n}$	
	R^2^	k	R^2^	k	R^2^	k	R^2^	n
	**DOX release**							
**MCM-41/DOX**	0.6538	0.0066	0.4695	0.0003	0.8252	1.2311	**0.9587**	0.6877
**MCM-41/DOX/Q**	0.6455	0.0065	0.4698	0.0002	0.8173	1.2205	**0.9758**	0.5376
**MCM-41-COOH/DOX**	0.7164	0.0069	0.4298	0.0004	0.8676	1.2668	**0.9971**	**0.4284**
**MCM-41-COOH/DOX/Q**	0.6993	0.007	0.4264	0.0004	0.8554	1.2829	**0.9982**	**0.4252**
	**Q release**							
**MCM-41/Q**	0.6416	0.0068	0.4204	0.0003	0.8159	1.2712	**0.9596**	0.6636
**MCM-41/DOX/Q**	0.6272	0.0068	0.4157	0.0003	0.8024	1.2823	**0.9798**	0.5185
**MCM-41-COOH/Q**	0.6852	0.0064	0.4124	0.0005	0.8470	1.1937	**0.9621**	0.5160
**MCM-41-COOH/DOX/Q**	0.6489	0.0062	0.3958	0.0004	0.8168	1.1652	**0.9770**	0.4737

**Table 5:** In vitro cytotoxicity (IC_50_ values) on cardioblast H9c2 cells of free DOX, free combination of non-loaded DOX + Q, non-carboxylated mesoporous silica nanoparticles single-loaded with DOX (MCM-41/DOX) and double-loaded with DOX and Q (MCM-41/DOX/Q), and carboxylated mesoporous silica nanoparticles single-loaded with DOX (MCM-41-COOH/DOX) and double-loaded with DOX and Q (MCM-41-COOH/DOX/Q) after 48 h treatment.

Treatment	IC_50_ (μM)	95% Confidence Interval
DOX	0.972	0.859 to 1.4851
DOX + Q	1.33	0.923 to 1.986
MCM-41/DOX	1.636	1.122 to 2.386
MCM-41/DOX/Q	3.028	2.236 to 4.102
MCM-41-COOH/DOX	2.756	1.849 to 3.264
MCM-41-COOH/DOX/Q	5.964	4.698 to 6.271

## Data Availability

Data are available from the authors (see given emails).
